# Genetic variation, linkage mapping of QTL and correlation studies for yield, root, and agronomic traits for aerobic adaptation

**DOI:** 10.1186/1471-2156-14-104

**Published:** 2013-10-29

**Authors:** Nitika Sandhu, Sunita Jain, Arvind Kumar, Balwant Singh Mehla, Rajinder Jain

**Affiliations:** 1 Division of Plant Breeding, Genetics, and Biotechnology, International Rice Research Institute, DAPO Box 7777 Metro Manila, Philippines; 2 Department of Biochemistry, CCS Haryana Agricultural University, Hisar 125004 India; 3 Department of Plant Breeding CCSHAU Rice Research Station, Kaul 136021 India; 4 Department of Molecular Biology and Biotechnology, CCS Haryana Agricultural University, Hisar 125004 India

**Keywords:** *Oryza sativa*, QTL, Rice, Aerobic, Dry direct seeded

## Abstract

**Background:**

Water scarcity and drought have seriously threatened traditional rice cultivation practices in several parts of the world, including India. Aerobic rice that uses significantly less water than traditional flooded systems has emerged as a promising water-saving technology. The identification of QTL conferring improved aerobic adaptation may facilitate the development of high-yielding aerobic rice varieties. In this study, experiments were conducted for mapping QTL for yield, root-related traits, and agronomic traits under aerobic conditions using HKR47 × MAS26 and MASARB25 × Pusa Basmati 1460 F_2:3_ mapping populations.

**Results:**

A total of 35 QTL associated with 14 traits were mapped on chromosomes 1, 2, 5, 6, 8, 9, and 11 in MASARB25 x Pusa Basmati 1460 and 14 QTL associated with 9 traits were mapped on chromosomes 1, 2, 8, 9, 10, 11, and 12 in HKR47 × MAS26. Two QTL (qGY_8.1_ with an R^2^ value of 34.0% and qGY_2.1_ with an R^2^ value of 22.8%) and one QTL (qGY_2.2_ with an R^2^ value of 43.2%) were identified for grain yield under aerobic conditions in the mapping populations MASARB25 × Pusa Basmati 1460 and HKR47 × MAS26, respectively.

A number of breeding lines with higher yield per plant, root length, dry biomass, length-breadth ratio, and with Pusa Basmati 1460-specific alleles in a homozygous or heterozygous condition at the *BAD2* locus were identified that will serve as novel material for the selection of stable aerobic Basmati rice breeding lines.

**Conclusions:**

Our results identified positive correlation between some of the root traits and yield under aerobic conditions, indicating the role of root traits for improving yield under aerobic situations possibly through improved water and nutrient uptake. Co-localization of QTL for yield, root traits, and yield-related agronomic traits indicates that the identified QTL may be immediately exploited in marker-assisted-breeding to develop novel high-yielding aerobic rice varieties.

## Background

Water scarcity is one of the most pressing issues facing agriculture today. In many countries, water for agriculture consumes about 70% of the total freshwater use. To meet the needs of a growing population, more food needs to be produced with less water [1]. Rice (*Oryza sativa* L.) is the primary source of food for more than half of the world’s population. Rice is cultivated in highly diverse situations that range from flooded wetland to rainfed dryland [2]. Irrigated rice, which accounts for 55% of the world rice area, provides 75% of global rice production and consumes about 90% of the freshwater resources used for agriculture in Asia [3]. Water deficit is a key constraint that affects rice production in different countries. The affinity of the rice crop with water is universally known. Conventional rice production ecosystems (puddled transplanted) require an average of 2,500 liters of water to produce 1 kg of rough rice [4], which is 2-3 times more than what other cereals require [5,6]. It is also reported that about 50% of the diverted fresh water in Asia is used to irrigate rice fields [5]. Seasonal water inputs for puddled transplanted rice vary from 660 to 5,280 mm depending on growing season, climatic conditions, soil type, and hydrological conditions [7]. Although evapo-transpirational losses of water in rice are similar to those in wheat [8], the higher water requirements for rice cultivation are mainly due to the water required for puddling, and high seepage and percolation losses associated with continuous flooding [9].

Rice can be established by transplanting seedlings in puddled fields or by direct seeding in dry or puddled fields [8]. Although the intensive water and labor requirements in transplanting of rice in puddled fields are well known, technologies such as dry and wet direct seeding and alternate wetting and drying (AWD) could be an option to produce rice in both irrigated and rainfed rice ecosystems. Aerobic rice is one such extensive water-saving technology for rice. Aerobic rice refers to a cultivation system in which rice is dry direct seeded in well-tilled leveled fields with uniform slope under unpuddled conditions. The crop is cultivated under aerobic conditions with no standing water throughout the season. In this system, rice can be established using different systems such as broadcasting, drilling, or dibbling in well-prepared fields and direct seeding with zero tillage using a mechanical seed drill or raised beds [8]. Traditionally, this method has been practiced in rainfed upland and rainfed shallow lowland areas of Asia [10]. However, the high proportion of water savings associated with this method compared with conventional rice-growing practices has made this method increasingly popular in irrigated areas where the problem of water shortage is also expanding [8]. Under favorable irrigated conditions, rice is drilled in well-prepared fields, and cultivated with efficient weed control and uniform irrigation throughout the crop season. Aerobic rice systems use less water than conventional flooded rice [6] through the use of rice varieties capable of responding well to reduced water inputs in non-puddled and non-saturated soils [11,12]. In the context of current and predicted water scarcity, increasing irrigation is generally not a viable option for alleviating drought problems in rainfed rice-growing systems [13]. It is therefore critical that genetic management strategies be undertaken for cultivating rice with less water and focusing on maximum extraction of available soil moisture and its efficient use in crop establishment and growth to maximize biomass and yield. With rice being grown extensively in the upland ecosystem, extensive genetic variation for aerobic adaptation exists in rice germplasm. However, the current challenge is to decipher the complexities of aerobic adaptation in rice and exploit all available genetic resources to produce rice varieties combining aerobic adaptation with high yield potential, good grain quality, and resistance to biotic stresses. This involves the development of high-throughput, high-precision phenotyping systems to allow genes for yield components under aerobic conditions to be efficiently mapped and their effects assessed for a range of traits, and then move the most promising genes into widely grown rice mega-varieties, while scaling up gene detection and delivery for use in marker-aided breeding.

One important aspect for understanding aerobic adaptation is the response of root growth and root development under aerobic conditions, including efficient uptake of water and nutrients. Roots are important for maintaining crop yields, which is vital when plants are grown in soils containing insufficient supplies of water or nutrients [14], and roots are one of the primary sites for stress signal perception that initiates a cascade of gene expression responses to water deficit [15]. Previous studies showed that plant growth largely depends on the severity of the stress; mild water deficit leads to growth inhibition of leaves and stems, whereas roots may continue to elongate [12]. Furthermore, root architecture is a key trait for dissecting the genotypic differences in rice responses to water deficit [16].

An understanding of the underlying physiological and molecular mechanisms is necessary to improve the adaptation of rice varieties to aerobic conditions. For water-deficit conditions, Price et al. [17] reported that a long and thick root system, the ratio of root weight to shoot weight, and root penetration ability of upland rice contribute greatly to drought tolerance. Several of the QTL identified for root length are consistent across mapping populations [18] and common genomic regions across populations and even across species have been identified for root thickness, root penetration, and stomatal behavior [19]. Progress has been made in detecting large-effect QTL conferring drought tolerance in lowland and irrigated rice [17,20]. Several QTL for grain yield under drought stress have been reported for both upland and lowland rice [21,22]. However, very few reports identify the genomic regions responsible for increased aerobic adaptation of rice. In our study, two aerobic × non-aerobic populations were studied for root traits, grain yield, and yield-related agronomic traits to identify QTL conferring a potential yield advantage under aerobic conditions; identify QTL for yield-related agronomic traits under aerobic conditions; identify root traits having a positive correlation with grain yield under aerobic conditions; identify QTL for root-related traits with a positive correlation with grain yield under aerobic conditions; and identify QTL for yield, yield-related agronomic traits, and root traits that co-localize with the aim to introgress identified QTL in HKR47 and Pusa Basmati 1460 rice varieties using marker-assisted breeding to develop rice varieties better adapted to aerobic conditions.

## Methods

Two mapping populations (HKR47 × MAS26 and MASARB25 × Pusa Basmati 1460) derived from crosses of HKR47 and Pusa Basmati 1460, lowland high-yielding rice cultivars unadapted to cultivation in aerobic conditions, with upland and aerobic adapted genotypes MAS26 (developed at the University of Agricultural Sciences, Bangalore) and MASARB25 (developed at IRRI, Manila, Philippines), respectively, were used in our study.

### Development and management of mapping populations

The F_2:3_ populations were developed from crosses involving MAS26 and MASARB25 as male parents and HKR47 and Pusa Basmati 1460 as female parents. True F_1_ seeds from the cross were grown to get F_2_ seeds. Seeds harvested from HKR47 × MAS26 and MASARB25 × Pusa Basmati 1460 F_1_ plants were germinated in petri plates and then a total of 235 and 250 germinated seeds from the two populations, respectively, were raised to maturity in pots (25 cm diameter × 25 cm height) with one plant/pot in a net house in 2010. The pots were irrigated with 1 liter of water for the first 15 days, and then with 1 liter after every third day up to panicle emergence. After every fifth day, the pots were irrigated with full-strength nutrient solution for the first 21 days, and with half-strength nutrient solution thereafter.

Seeds obtained from each F_2_ plant were evaluated in fields at the Rice Research Station, Kaul, Karnal, India, during 2011 and 2012 under dry direct-seeded aerobic cultivation practices that involved dry seeding at approximately 2-cm depth in dry-ploughed and harrowed aerobic plots with row spacing of 30 cm (2011) and 25 cm (2012), resulting in a seed rate of approximately 305 seeds m^-2^ in 2011 and 365 seeds m^-2^ in 2012. The seeds obtained from each selected F_2_ plant (20% best and 20% worst, 94 plants in the case of HKR47 × MAS26 and 100 plants in the case of MASARB25 × Pusa Basmati 1460) were grown in 2-m single-row plots with two replications using a seeding density of 2 g per linear meter of row to record observations for the root study, grain yield, agronomic traits, and genotyping. For the yield trial, the plots were randomized by using Crop Stat version 7.2. Aerobic fields were irrigated for about 1 week with a 2-3-cm water layer to facilitate crop establishment; thereafter, the fields were re-irrigated once at a 10-day interval. Nitrogen was applied at 60 kg ha^-1^ basal after sowing, 60 kg ha^-1^ 25 days after sowing, and 60 kg ha^-1^ 55 days after sowing. In addition, 30 kg P ha^-1^, 40 kg K ha^-1^, and 5 kg Zn ha^-1^ were applied as basal and 30 kg P ha^-1^ at 25 days after sowing. The plots were kept weed-free by manual weeding. At physiological maturity or 80-85% of the panicles turned into golden yellow and the panicles at the base were already at hard dough stage, data were recorded on agronomic traits, plant height in cm (PH), effective number of tillers per plant (TN), panicle length in cm (PL), number of panicles per plant (P/P), number of grains per panicle (S/P), 1,000-grain weight (1,000 GW), length/breadth ratio (L/B), and grain yield in Kg ha^-1^ (GY). Grain yield per plot was recorded after harvesting, threshing, and drying to moisture content adjusted to 14% and converted to kg ha^-1^. The data on root morphological traits—root length (RL, cm), root thickness (RT), root number (RN), root volume (RV), fresh and dry root weight (FRW and DRW in g), and fresh and dry shoot weight (FSW and DSW in g) from six plants from each line at maturity were recorded and analyzed. For the measurement of root traits, plants were removed from the soil. Soil sections that contained roots were removed by digging a hole and put into a sieve and gently washed with a hose until only the roots remained. The roots and shoots were then separated by cutting from the top soil line. For fresh root weight, the roots were blotted gently with a soft paper towel to remove any free surface moisture. Then, the roots and shoots were weighed immediately (plants have a high composition of water, so waiting to weigh them may lead to some drying and therefore produce inaccurate data). Root volume was measured by the actual volume displacement analysis, which measures the volume of water displaced when roots are submerged in a vessel of water. Volume displacement analysis has the advantage of providing a fast measure of new root production. In addition, its non-destructive nature permits repeated measures over time. The thickness of the root crown was measured using a vernier caliper. Root numbers were counted manually. Root length was measured using a centimeter scale. For dry root weight, the roots and shoots were dried in an oven set to low heat (100°F) for 3 days, and then cooled in a dry environment. Once cooled, they were weighed on a scale.

### Statistical analysis

Statistical analysis of the data on individual characters was performed using SAS v9.1 (SAS Institute, Inc., 2002-2003). Line means were estimated using the REML option of the SAS MIXED procedure taking lines as fixed and replicates and blocks within replicates as random. The phenotypic r(P) correlation coefficients for all possible pairs of characters were calculated from the variance and covariance was estimated:

$$r\left(P\right)=\sigma \mathrm{xy}\left(P\right)/\left[\sigma x\left(P\right)X\sigma y\left(P\right)\right]$$

where

σ x y (P) = Phenotypic covariance between characters x and y

σ^2^ x (P) = Phenotypic variance of character x

σ^2^ y (P) = Phenotypic variance of character y

The phenotypic correlation coefficients were tested against a standardized tabulated significant value of r with (n-2) degree of freedom as per the procedure of Fisher and Yates [23].

### Genotyping of mapping populations

All DNA marker work was conducted in the Department of Molecular Biology and Biotechnology, CCSHAU, Hisar, India. Six fresh leaves from each line were collected in bulk and genomic DNA was isolated from 1-month-old plants using the CTAB method [24]. DNA quantity was estimated by ethidium bromide staining on 1% agarose gels using a standard containing 100 ng/μl genomic DNA. PCR amplification, denaturing polyacrylamide gel electrophoresis, and silver staining were essentially carried out as described earlier by Jain et al. [25].

### Genetic analysis

A total of 300 mapped SSR and aroma gene-specific *BAD2* markers (for which basmati serves as a parent) were screened for polymorphism between the parents. The markers were obtained based on published rice genome maps (IRGSP 2005) and their physical position (Mb) on the *indica* genome (http://www.gramene.org) was used as a reference. A total of 91 and 112 markers showed polymorphism and were run on 94 and 100 plants for HKR47 x MAS26 and MASARB25 × Pusa Basmati 1460 populations, respectively. Genetic similarities between the cultivars were measured by the similarity coefficient based on the proportion of shared electromorphs using the 'Simqual’ subprogram of the NTSYS-PC (Version 2.02 Exeter Software, Setauket, NY, USA) software package [26]. The resultant distance matrix data were used for two-dimensional scaling of rice genotypes by principal component analysis (PCA). Linkage analysis was performed using the Map Manager/QTX computer program [27] using the Kosambi function and linkage evaluation of P = 0.001. The ripple command was used to verify the marker order. QTL analysis was performed using Windows QTL Cartographer version 2.5 [28]. For interval mapping (IM) analysis, an LOD threshold score of 2.5 was selected. The proportion of the total phenotypic variation explained by each QTL was calculated as R^2^ value (R^2^ = ratio of the sum of squares explained by the QTL to the total sum of squares). To more accurately determine QTL positions, composite interval mapping (CIM) was performed with default parameters (permutation time 300, significance level 0.05, model 6; standard model, method 3; forward and backward method, walk speed 2 cM, etc.).

## Results

### Phenotypic analysis of parents and populations

MAS26 and MASARB25, the aerobic genotypes, outyielded the lowland parents, HKR47 and Pusa Basmati 1460, in both years under dry direct-seeded aerobic conditions. Root length, root volume, root thickness, root number, and root biomass of the tolerant parent were found to be higher than those of the susceptible parent. Mean values of RL, RV, RT, RN, FRW, DRW, FSW, and DSW for the two populations are presented in Table 1. Mean and range values of PH, TN, PL, P/P, S/P, 1,000 GW, and GY on two populations under dry direct-seeded aerobic conditions in 2011 and 2012 are presented in Table 2.

**Table 1:** Mean and range for various root traits in two populations under pot-house conditions

**Trait**	**HKR47**	**MAS26**	**MASARB25**	**Pusa Basmati 1460**	**HKR47 × MAS26**		**MASARB25 × Pusa Basmati 1460**	
					**Mean**	**Range**	**Mean**	**Range**
RL	26 ± 0.784	39.45 ± 0.958	32.43 ± 0.975	21.89 ± 1.156	27.54	17-49	29.46	12-48
RN	92 ± 1.172	97 ± 1.059	110.0 ± 2.045	92.0 ± 1.685	83.3	34-220	98	35-222
RV	7.3 ± 0.497	8.9 ± 0.677	15.0 ± 0.779	12.0 ± 0.931	8.0	4-18	10.1	3-22
RT	2.4 ± 0.884	2.9 ± 0.679	3.72 ± 0.553	2.98 ± 0.478	2.85	1.8-4.4	3.27	2.1-5.6
FRW	2.64 ± 0.948	3.34 ± 0.869	4.88 ± 0.585	3.79 ± 0.664	3.08	1.30-6.39	4.04	1.34-11.13
FSW	23.36 ± 1.558	26.18 ± 1.037	28.77 ± 1.156	23.39 ± 1.473	22.27	15.32-46.44	25.88	16.14-56.34
DRW	1.22 ± 0.447	0.89 ± 0.375	1.56 ± 0.661	1.13 ± 0.449	0.92	0.24-2.94	1.22	0.22-3.62
DSW	10.18 ± 0.836	11.99 ± 0.991	12.74 ± 0.868	10.89 ± 0.921	9.18	3.42-19.72	12.13	4.34-35.26

*RL*: Root length (cm), *RN*: Root number, *RV*: Root volume (ml), *RT*: Root thickness (mm), *FRW*: Fresh root weight (g), *FSW*: Fresh shoot weight (g), *DRW*: Dry root weight (g), *DSW*: Dry shoot weight (g).

**Table 2:** Mean and range for various agronomic traits in two populations under direct-seeded aerobic field conditions

**Trait**	**HKR47**		**MAS26**		**MASARB25**		**Pusa Basmati 1460**		**HKR47 × MAS26**				**MASARB25 × Pusa Basmati 1460**			
									**Mean**		**Range**		**Mean**		**Range**	
	**2011**	**2012**	**2011**	**2012**	**2011**	**2012**	**2011**	**2012**	**2011**	**2012**	**2011**	**2012**	**2011**	**2012**	**2011**	**2012**
PH	82	86.3	71	78.0	75	82	80	103	75.9	81.9	63-92	71-101	70.6	81.2	50-85	66-97
TN	18	15	21	18	19	21	15	12	13	16	3-30	8-28	13.88	16.22	9-28	11-24
P/P	16	13	20	15	16	19	13	11	12	14	3-26	5-25	11	15	7-24	6-21
S/P	74	80	88	91	75	80	82	89	58	65	48-109	40-100	62	55	43-107	32-99
GY	820	747	972	932	908	852	788	717	760	834	302-1450	358-1222	702	878	401-1598	323-1410
1,000 GW	27.5	22.4	29.4	23.4	29.4	35.9	28.3	8.8	30.7	23.3	22.7-59.7	30.0-51.1	33.8	39.9	25.3-54.0	20.2-64.2
PL	20.8	21.7	17.4	19.5	18.4	20.9	20.9	22.5	19.1	21.3	14.4-25.5	18.8-27.5	19.0	17.7	14.8-23.1	15.5-28.6
L/B	4.15	3.89	4.34	4.24	4.66	4.27	5.23	4.91	4.20	3.29	3.89-4.73	3.31-4.44	5.09	4.99	4.30-5.84	4.77-6.12

*PH*: Plant height (cm), *TN*: Effective number of tillers plant^-1^, *P/P*: No. of panicles plant^-1^, *S/P*: Seeds panicle^-1^, *GY*: Grain yield (Kg ha^-1^), *1,000 GW*: 1,000-grain weight (g), *PL*: Panicle length (cm), L/B: Length/Breadth ratio.

In both mapping populations, grain yield was found to be positively correlated with root length, root number, root volume, and root biomass, suggesting that these traits might be the key factor for improving grain yield under aerobic conditions (Table 3).

**Table 3:** Phenotypic correlation coefficients between agronomic and root traits in MASARB25 × Pusa Basmati and HKR47 × MAS26-derived populations

		**L/B**	**P/P**	**PH**	**PL**	**S/P**	**TN**	**GY**	**DRW**	**DSW**	**FRW**	**FSW**	**RL**	**RN**	**RT**	**RV**
MASARB25 × Pusa Basmati 1460	L/B	1														
	P/P	0.404*	1													
	PH	0.277*	0.461**	1												
	PL	0.354**	0.420**	0.397**	1											
	S/P	0.202	0.494**	0.265*	0.604**	1										
	TN	0.415**	0.975**	0.485**	0.426**	0.476**	1									
	GY	0.689**	0.427**	0.297*	0.411**	0.377**	0.442**	1								
	DRW	0.301*	0.222	0.235	0.488**	0.241*	0.210	0.330**	1							
	DSW	0.063	0.352**	0.125	0.266*	0.310**	0.311**	0.246*	0.648**	1						
	FRW	0.306*	0.305*	0.130	0.451**	0.336**	0.297*	0.379**	0.782**	0.612**	1					
	FSW	0.152	0.313**	0.090	0.245*	0.335**	0.276*	0.340**	0.651**	0.918**	0.663**	1				
	RL	0.146	0.000	-0.078	0.258*	0.245*	0.013	0.235*	0.281*	0.392**	0.397**	0.417**	1			
	RN	0.106	0.365**	0.101	0.324**	0.367**	0.337**	0.285*	0.575**	0.867**	0.630**	0.857**	0.602**	1		
	RT	0.130	0.335**	0.134	0.337**	0.349**	0.281*	0.278*	0.625**	0.775**	0.544**	0.745**	0.352**	0.770**	1	
	RV	0.172	0.279*	0.241*	0.358**	0.295*	0.261*	0.265*	0.575**	0.606**	0.621**	0.586**	0.700**	0.741**	0.587**	1
HKR47 × MAS26	L/B	1														
	P/P	0.105	1													
	PH	0.044	0.492**	1												
	PL	0.259	0.457**	0.431**	1											
	S/P	0.212	0.529**	0.422**	0.694**	1										
	TN	0.113	0.997**	0.480**	0.460**	0.532**	1									
	GY	0.725**	0.294*	0.330*	0.512**	0.476**	0.296*	1								
	DRW	0.101	0.468**	0.404**	0.462**	0.557**	0.466**	0.352**	1							
	DSW	0.187	0.505**	0.205	0.530**	0.552**	0.507**	0.377**	0.569**	1						
	FRW	0.126	0.545**	0.419**	0.514**	0.573**	0.539**	0.346**	0.834**	0.699**	1					
	FSW	0.155	0.452**	0.200	0.384**	0.429**	0.453**	0.221	0.494**	0.878**	0.681**	1				
	RL	0.239	0.297*	-0.059	0.324*	0.355**	0.289	0.317*	0.285*	0.662**	0.353**	0.521**	1			
	RN	0.270*	0.697**	0.384**	0.371**	0.417**	0.695**	0.386**	0.449**	0.517**	0.482**	0.419**	0.413**	1		
	RT	0.225	0.640**	0.239	0.482**	0.554**	0.628**	0.408**	0.485**	0.718**	0.626**	0.551**	0.475**	0.746**	1	
	RV	0.322*	0.636**	0.296*	0.441**	0.468**	0.63**	0.455**	0.442**	0.614**	0.492**	0.531**	0.533**	0.810**	0.728**	1

*L/B*: Length/breadth ratio, *P/P*: No. of panicles/plant, *PH*: Plant height, *PL*: Panicle length, *TN*: Tiller number, *GY*: Grain yield, *DRW*: Dry root weight, *DSW*: Dry shoot weight, *FRW*: Fresh root weight, *FSW*: Fresh shoot weight, *RL*: Root length, *RN*: Root number, *RT*: Root thickness, *RV*: Root volume, *Correlation is significant at 0.05 level, **Correlation is significant at 0.01 level.

Phenotypic correlation coefficient analysis of MASARB25 × Pusa Basmati 1460 showed that GY is positively and significantly correlated with the effective number of tillers (0.442, p = 0.01), panicle length (0.411, p = 0.01), panicles plant^-1^ (0.427, p = 0.01), seeds panicle^-1^ (0.377, p = 0.01), and length/breadth ratio (0.689, p = 0.01). In this population, root number is positively and significantly correlated with panicles plant^-1^ (0.365, p = 0.01), panicle length (0.324, p = 0.01), seeds panicle^-1^ (0.367, p = 0.01), GY (0.285, p = 0.05), dry root weight (0.575, p = 0.01), fresh root weight (0.630, p = 0.01), and root length (0.602, p = 0.01). Root thickness has been found to be positively and significantly correlated with fresh root weight (0.544, p = 0.01), root length (0. 0.352, p = 0.01), root number (0.770, p = 0.01), dry root weight (575, p = 0.01), panicles plant^-1^ (0.335, p = 0.01), and GY (0.278, p = 0.05). Root volume is positively and significantly correlated with panicle length (0.358, p = 0.01), dry root weight (0.575, p = 0.01), fresh root weight (0.621, p = 0.01), root thickness (0.587, p = 0.01), root number (0.740, p = 0.01), and root length (0.700, p = 0.01). Dry root weight is positively and significantly correlated with GY (0.330, p = 0.01) and seeds panicle^-1^ with panicle length (0.604, p = 0.01). Root length was found to be negatively correlated with plant height (Table 3).

Phenotypic correlation coefficient analysis of HKR47 × MAS26 showed that GY is positively and significantly correlated with panicle length (0.512, p = 0.01), panicles plant^-1^ (0.294, p = 0.05), seeds panicle^-1^ (0.476, p = 0.01), and length/breadth ratio (0.725, p = 0.01). In this population, seeds panicle^-1^ is positively and significantly correlated with panicle length (0.694, p = 0.01) and panicles plant^-1^ (0.529, p = 0.01). Root number is positively and significantly correlated with GY (0.386, p = 0.05) and fresh root weight (0.482, p = 0.01). Root thickness has been found to be positively and significantly correlated with fresh root weight (0.626, p = 0.01), root number (0.746, p = 0.01), and GY (0.408, p = 0.01). Root volume is positively and significantly correlated with fresh root weight (0.412, p = 0.01), root thickness (0.728, p = 0.01), and GY (0.455, p = 0.01). Root length is positively and significantly correlated with GY (0.317, p = 0.05) and seeds panicle^-1^ (0.355, p = 0.01). Fresh root weight is positively and significantly correlated with panicles plant^-1^ (0.412, p = 0.01), GY (0.346, p = 0.01), and effective number of tillers (0.539, p = 0.01) (Table 3).

### Genotypic analysis of mapping populations

Polymorphism of HKR47 with MAS26 and of MASARB25 with Pusa Basmati 1460 was 31.7% and 37.3%, respectively. A total of 226 and 184 alleles were identified in the HKR47 × MAS26 and MASARB25 Pusa Basmati 1460 populations by using 300 SSR markers, respectively. MASARB25 × Pusa Basmati 1460-derived mapping populations were also evaluated for aroma using aroma gene-specific *BAD2* markers. A total of 13 lines were found to be homozygous and 24 lines were found to be heterozygous for aroma.

**Figure 1:**
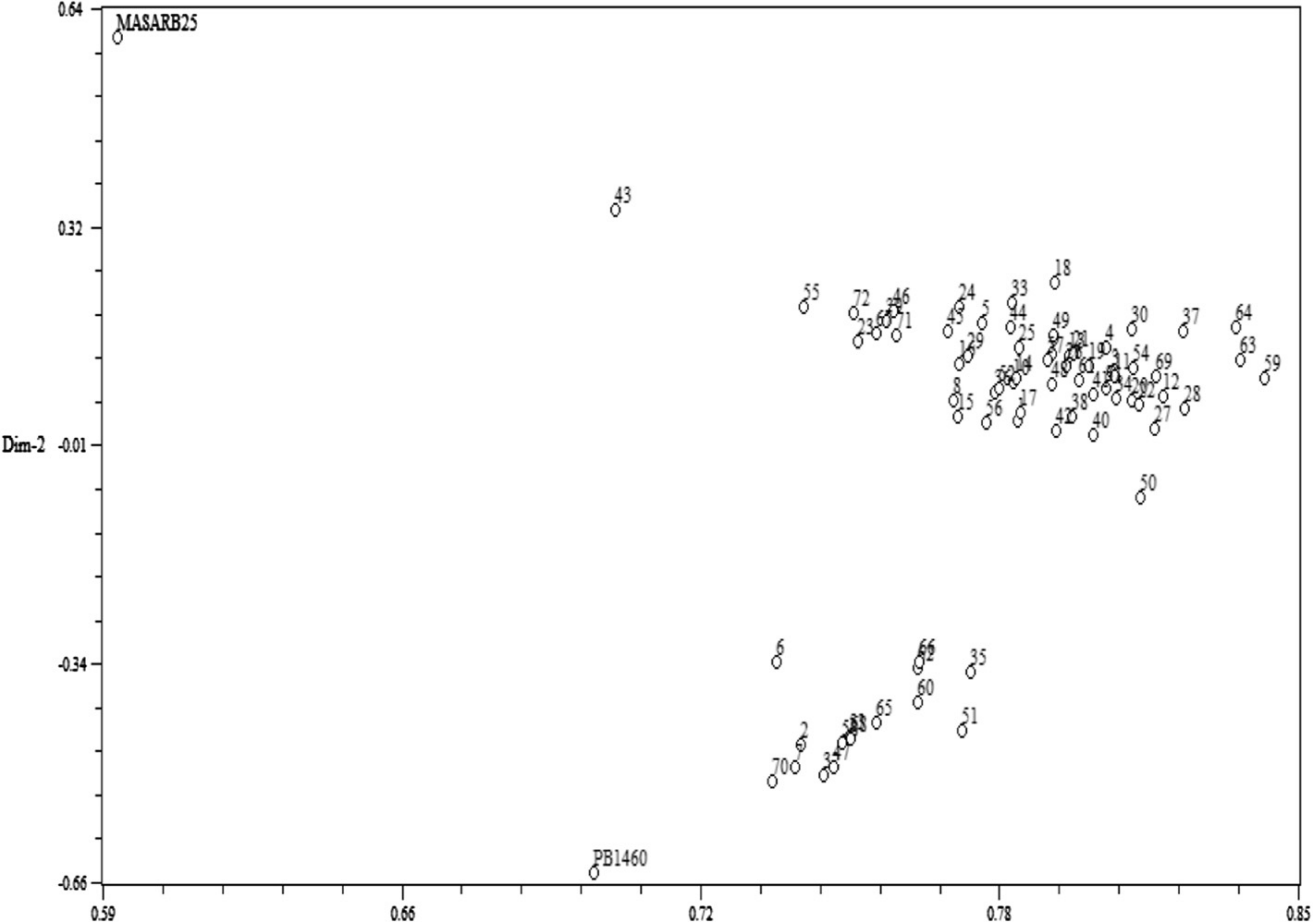
Two-dimensional PCA scaling of MASARB25 × Pusa Basmati 1460 based on SSR diversity data.

**Figure 2:**
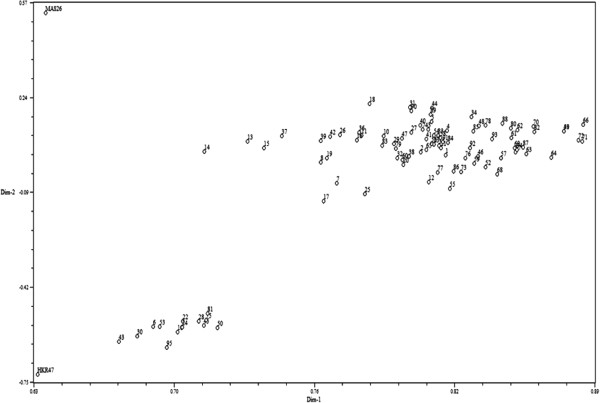
Two-dimensional PCA scaling of HKR47 × MAS26 based on SSR diversity data.

Similarity coefficient data based on the proportion of shared alleles using 91 and 112 SSR markers were used to calculate the coefficient values among the selected HKR47 × MAS26 and MASARB25 × Pusa Basmati 1460 lines, respectively, through UPGMA tree cluster analysis. Allelic diversity was used to produce a dendrogram (cluster tree analysis, NTSYS-PC) to demonstrate the genetic relationship among selected plants and the parental rice varieties. All 94 and 100 plants from the two populations, HKR47 × MAS26 and MASARB25 × Pusa Basmati 1460, respectively, clustered in two major groups at the similarity coefficients of 0.520 and 0.540, respectively. Genetic relationship was also assessed by PCA analysis (NTSYS-PC). Two-dimensional PCA scaling showed that the parental genotypes were quite distinct, whereas the mapping population genotypes were interspersed between the two parental lines (Figures 1 and 2).

Several QTL associated with root traits and agronomically important traits in the two mapping populations were detected in the MASARB25 x Pusa Basmati 1460 (Table 4) and HKR47 × MAS26 (Table 5) populations. A total of 35 QTL associated with 14 traits were mapped on chromosomes 1, 2, 5, 6, 8, 9, and 11 in MASARB25 × Pusa Basmati 1460-derived population (Table 4, Additional file 1: Figure S1). A total of 14 QTL associated with nine traits were mapped on chromosomes 1, 2, 8, 9, 10, 11, and 12 in HKR47 × MAS26-derived population (Table 5, Additional file 1: Figure S2).

**Table 4:** QTL associated with agronomic and aerobic root traits of MASARB25 × Pusa Basmati 1460 population

**Trait**	**Name**	**Chr. no.**	**Position (cM)**	**Flanking markers**	**Position of flanking markers (cM)**	**LR**	**LOD**	**Additive effect**	**R**^**2**^	**DPE**^**a**^
Root length	qRL_8.1_	8	36.5	RM408-RM544	0.6-38.5	21.51	4.6	-3.244	25.1	P
	qRL_8.2_	8	58.3	RM547-RM331	58.1-65.0	33.35	7.2	4.066	38.8	M
Root volume	qRV_2.1_	2	0.8	RM485-RM7033	0.0-7.9	16.8	3.7	2.492	19.7	M
	qRV_6.1_	6	20.3	RM587-RM204	10.7-25.1	11.7	2.5	-2.349	18.1	P
Root thickness	qRT_5.1_	5	6.7	RM122-RM574	6.4-41.0	15.04	3.3	0.375	22.3	M
	qRT_6.1_	6	26.0	RM204-RM225	25.1-26.2	14.40	3.1	-0.33	17.4	P
Fresh root weight	qFRW_2.1_	2	2.5	RM485-RM7033	0.0-7.9	17.27	3.8	1.194	28.9	M
	qFRW_2.2_	2	60.0	RM174-RM300	47.5-66.0	12.11	2.6	-1.049	24.6	P
	qFRW_6.1_	6	3.4	RM589-RM587	3.2-10.7	11.71	2.5	-0.901	17.2	P
Dry root weight	qDRW_2.2_	2	48.1	RM174-RM300	47.5-66.0	12.5	2.7	-0.298	18.4	P
	qDRW_8.2_	8	123.0	RM458-RM3331	122.9-123.2	14.1	3.1	-0.294	18.1	P
Fresh shoot weight	qFSW_2.1_	2	6.1	RM485-RM7033	0.0-7.9	13.8	3.0	5.204	20.6	M
	qFSW_6.2_	6	28.5	RM225-RM314	26.2-33.6	22.6	4.9	-6.020	31.4	P
	qFSW_6.1_	6	21.1	RM587-RM204	10.7-25.1	18.1	3.9	-5.730	30.5	P
Dry shoot weight	qDSW_2.1_	2	5.8	RM485-RM7033	0.0-7.9	11.9	2.6	3.260	23.1	M
	qDSW_6.1_	6	25.1	RM204	25.1	18.7	4.1	-3.510	29.4	P
Plant height	qPH_2.1_	2	2.2	RM485-RM7033	0.0-7.9	14.5	3.0	14.450	24.3	M
	qPH_6.1_	6	56.2	RM6836-RM527	54.1-61.2	12.3	2.7	12.340	18.9	M
	qPH_9.1_	9	56.1	RM524-RM410	13.2-64.4	21.49	4.7	-8.330	67.7	P
	qPH_11.2_	11	99.8	RM21-RM206	85.7-102.9	17.81	3.9	-4.580	21.0	P
Panicle length	qPL_2.1_	2	4.4	RM485-RM7033	0.0-7.9	20.92	4.5	2.030	28.6	M
	qPL_11.1_	11	39.0	RM4-RM441	0.0-43.9	15.68	3.4	-1.820	26.4	P
	qPL_11.2_	11	48.1	RM441-RM21	43.9-85.7	16.59	3.6	-1.860	28.0	P
	qPL_8.1_	8	112.8	RM230-RM433	112.2-116.0	25.24	5.4	1.260	16.5	M
	qPL_9.1_	9	56.2	RM524-RM410	13.2-64.4	17.15	3.7	-2.050	64.2	P
Tiller number	qTN_2.1_	2	152.7	RM1092-RM318	146.6-152.8	11.97	2.6	-0.979	14.5	P
	qTN_1.1_	1	86.9	RM10916-RM246	67.4-115.2	13.77	3.0	-2.340	20.3	P
	qTN_8.2_	8	58.3	RM547-RM331	58.1-65.0	20.32	4.4	-3.361	47.9	P
Panicles plant^-1^	qNPP_2.1_	2	153.0	RM318-RM6	152.8-154.7	12.05	2.6	-0.979	14.5	P
	qNPP_8.2_	8	58.4	RM547-RM331	58.1-65.0	1.64	6.4	-0.947	13.7	P
Seeds panicle^-1^	qNSP_11.1_	11	37.5	RM4-RM441	0.0-43.9	14.85	3.2	-10.840	38.9	P
	qNSP_11.2_	11	47.8	RM441-RM21	43.9-85.7	14.02	3.0	-10.060	34.7	P
Length/breadth ratio	qLBR_11.1_	11	55.1	RM441-RM21	43.9-85.7	14.53	3.2	-0.277	39.1	P
Grain yield	qGY_8.1_	8	72.2	RM339	72.2	21.54	4.7	-199.200	34.0	P
	qGY_2.1_	2	10.2	RM7562-RM279	8.6-13.8	13.74	3.0	147.250	22.8	M

^a^Direction of phenotypic effect; *M* and *P* indicate MASARB25 and Pusa Basmati 1460 alleles, respectively. Additive effect is the effect of substituting a MASARB25 allele for a Pusa Basmati 1460 allele; Its positive value indicates that MASARB25 has the positive allele; *LR:* Likelihood ratio, *LOD:* log_10_ of an odds ratio, *R*^*2*^*:* percent phenotypic variance.

**Table 5:** QTL associated with agronomic and aerobic root traits of HKR47 × MAS26 population

**Trait**	**Name**	**Chr. no.**	**Position (cM)**	**Flanking markers**	**Position of flanking markers (cM)**	**LR**	**LOD**	**Additive effect**	**R**^**2**^	**DPE**^**a**^
Root length	qRL_8.2_	8	57.8	RM310-RM547	57.0-58.1	13.58	2.9	1.980	19.8	M
	qRL_9.1_	9	34.1	RM524-RM257	13.2-66.1	11.87	2.6	-2.800	31.6	H
Root thickness	qRT_1.1_	1	107.9	RM488-RM237	101.4-112.9	20.43	4.4	0.406	38.4	M
Dry root weight	qDRW_8.1_	8	14.6	RM152-RM310	9.4-57.0	21.26	4.6	-0.439	44.7	H
Plant height	qPH_8.1_	8	101.5	RM256	101.5	16.00	3.5	-4.424	28.2	H
	qPH_11.1_	11	11.9	RM4-RM202	5.0-6.2	16.10	3.5	-5.682	42.6	H
Panicle length	qPL_12.1_	12	47.4	RM28048	47.4	13.40	2.9	1.952	36.8	M
	qPL_10.1_	10	70.8	RM258	70.8	11.78	2.6	1.200	21.6	M
Tiller number	qTN_8.3_	8	123.2	RM3331	123.2	16.60	3.6	-1.730	26.4	H
	qTN_8.1_	8	15.7	RM152-RM310	9.4-57.0	11.90	2.6	-3.124	29.3	H
Panicles plant^-1^	qNPP_8.3_	8	123.2	RM3331	123.2	13.82	3.0	-1.340	20.2	H
	qNPP_8.1_	8	16.2	RM152-RM310	9.4-57.0	13.51	2.9	-2.790	31.6	H
Seeds panicle^-1^	qNSP_8.1_	8	123.2	RM3331	123.2	11.46	2.5	-186.300	15.3	H
Grain yield	qGY_2.2_	2	118.5	RM475-RM526	92.5-136.3	12.56	2.7	145.100	43.2	M

^a^Direction of phenotypic effect; *H* and *M* indicate HKR47 and MAS26 alleles, respectively. Additive effect is the effect of substituting a MAS26 allele for an HKR47 allele; its positive value indicates that MAS26 has the positive allele; *LR:* Likelihood ratio, *LOD:* log_10_ of an odds ratio, *R*^*2*^*:* percent phenotypic variance.

QTL qRL_8.1_ with a peak at RM544 on chromosome 8 with an R^2^ value of 25.1% and qRL_8.2_ with a peak at RM547 on chromosome 8 with an R^2^ value of 38.8% and QTL qRL_8.2_ with a peak at RM547 on chromosome 8 with an R^2^ value of 19.8% and qRL_9.1_ with a peak at RM524 on chromosome 9 with an R^2^ value of 31.6% were identified associated with root length in the mapping populations MASARB25 × Pusa Basmati 1460 and HKR47 × MAS26, respectively. qRL_8.2_ was identified in both populations.

**Figure 3:**
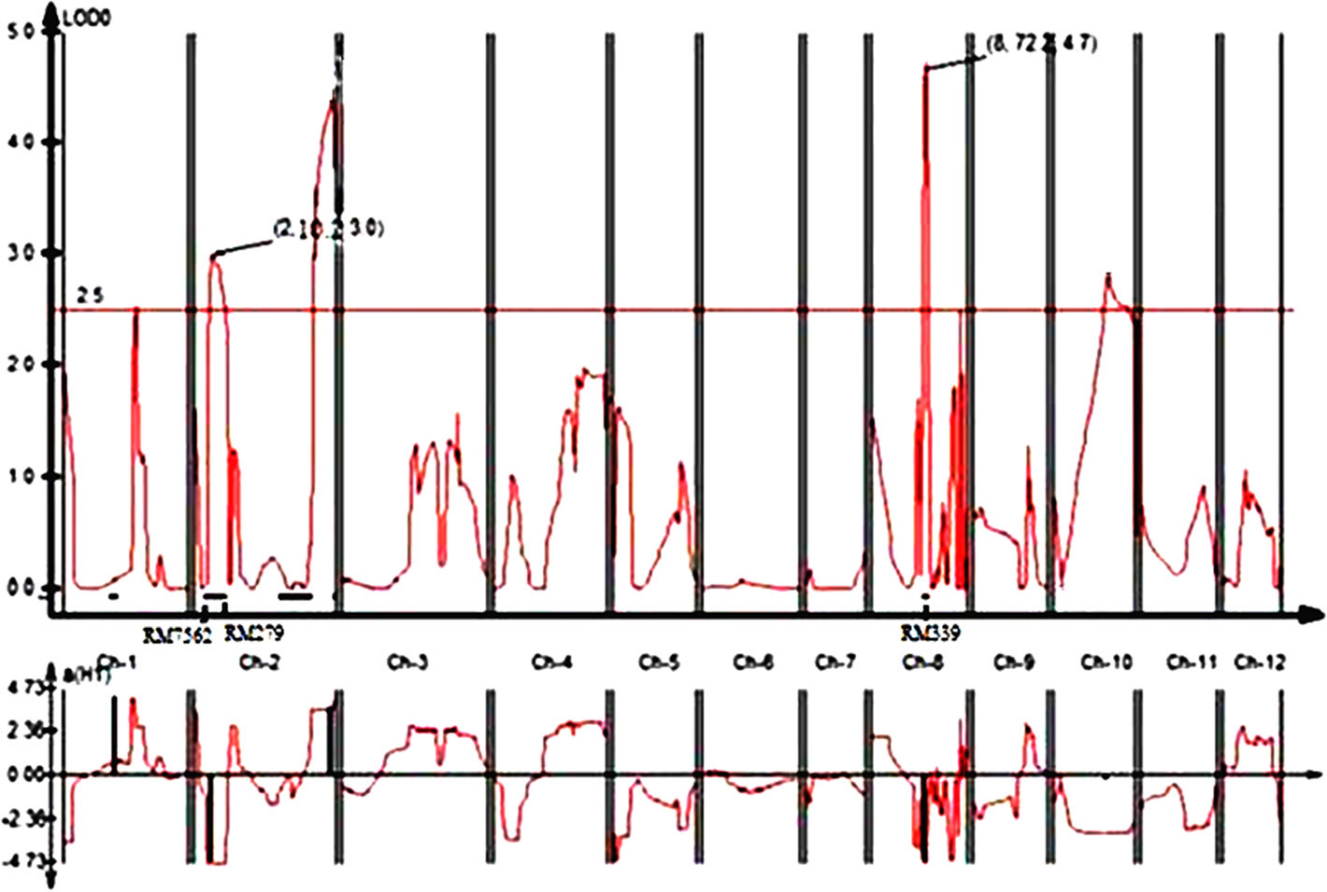
**QTL likelihood curves of LOD scores for grain yield showing significant regions within qGY**_**2.1 **_**and qGY**_**8.1 **_**under aerobic conditions in MASARB25 × Pusa Basmati 1460.**

**Figure 4:**
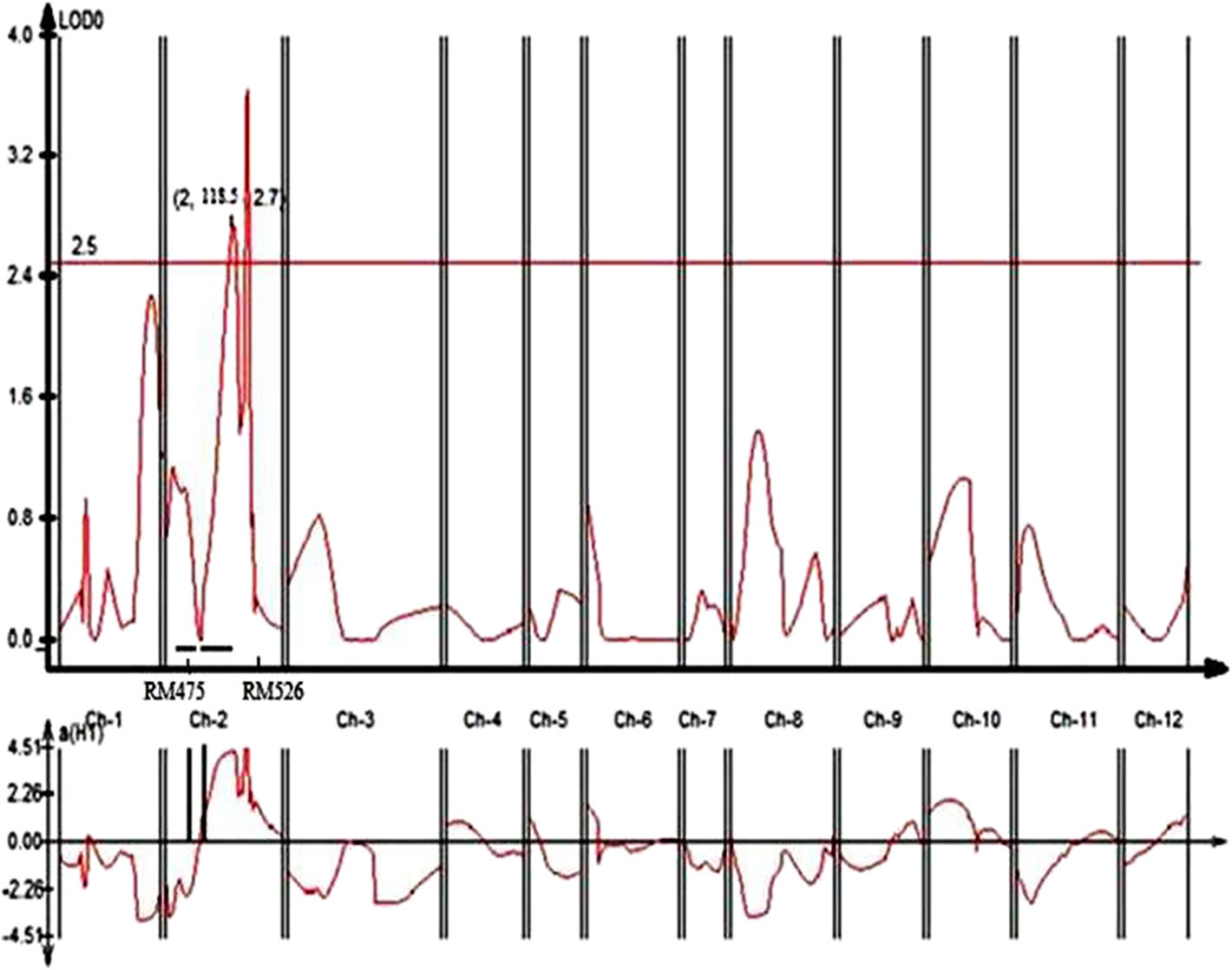
**QTL likelihood curves of LOD scores for grain yield showing significant regions within qGY**_**2.2 **_**under aerobic conditions in HKR47 × MAS26.**

In addition, QTL qGY_8.1_ with RM339 on chromosome 8 being the peak marker with an R^2^ value of 34.0% and qGY_2.1_ on chromosome 2 with RM7562 being the peak marker with an R^2^ value of 22.8% (Figure 3) and QTL qGY_2.2_ on chromosome 2 with RM526 being the peak marker with an R^2^ value of 43.2% (Figure 4) were identified for grain yield under aerobic conditions in the mapping populations MASARB25 × Pusa Basmati 1460 and HKR47 × MAS26, respectively.

For the MASARB25 × Pusa Basmati 1460 population on chromosome 2 in a region of 0-10.2 cM, seven QTL were reported to be significantly associated with seven different root traits and agronomically important traits (RV, FRW, FSW, DSW, PH, PL, and GY). These coexisting chromosomal regions governing root and yield traits provide a unique opportunity for breeders to introgress such regions together as a unit into lowland varieties through MAS/MAB and to develop cultivars possessing higher yield and increased adaptation to aerobic conditions.

In HKR47 × MAS26-derived mapping population, RM256 on chromosome 8, RM28048 on chromosome 12 and RM258 on chromosome 10, and RM3331 on chromosome 8 showed significant association with plant height, panicle length, and number of tillers per plant, respectively (Table 5). RM339 on chromosome 8 and RM204 on chromosome 6 showed significant association with grain yield and dry shoot weight in MASARB25 × Pusa Basmati 1460-derived mapping population. On chromosome 2 in a region of 0-10.2 cM, seven QTL were reported to be significantly associated with seven different root traits and agronomically important traits in the case of MASARB25 × Pusa Basmati 1460 population (Table 4).

In this study, three QTL for root thickness (chromosomes 1, 5, and 6), two for root volume (chromosomes 2 and 6), three for fresh root weight (chromosomes 2 and 6), three for dry root weight (chromosomes 2 and 8), three for fresh shoot weight (chromosomes 2 and 6), two for dry shoot weight (chromosomes 2 and 6), six for plant height (chromosomes 2, 6, 8, 9, and 11), seven for panicle length (chromosomes 2, 8, 9, 10, 11, and 12), five for tiller number (chromosomes 1, 2, and 8), four for number of panicles per plant (chromosomes 2 and 8), three for number of seeds per panicle (chromosomes 8 and 11), one for length/breadth ratio (chromosome 11), and three for grain yield (chromosomes 2 and 8) were identified (Tables 4 and 5). qGY_2.1_ and qGY_8.1_, individually and combined, and qGY_2.2_ exhibited a grain yield improvement of 24%, 28%, 37%, and 36%, respectively, in 2011 and 18%, 25%, 39%, and 24%, respectively, in 2012 under aerobic conditions (Table 6).

**Table 6:** Yield improvement of lines possessing QTL (QTL+) for grain yield under aerobic conditions over lines not possessing QTL (QTL-) for the two populations

**Trial**	**QTL**	**+QTL**	**-QTL**	**PB1460/HKR47**	**% Yield improvement**
MASARB25 × Pusa Basmati 1460					
2011	qGY_2.1_	1255.3	1014.9	788	24
	LSD_0.05_	163.4	131.6	126.6	
	qGY_8.1_	1352.3	1057.0	788	28
	LSD_0.05_	170.2	126.2	126.6	
	qGY_2.1_ and qGY_8.1_	1500.3	1094.8	788	37
	LSD_0.05_	195.4	141.0	126.6	
2012	qGY_2.1_	1087.2	922.9	717	18
	LSD_0.05_	134.6	112.2	104.2	
	qGY_8.1_	1237	987.2	717	25
	LSD_0.05_	151.2	124.2	104.2	
	qGY_2.1_ and qGY_8.1_	1317	949.5	717	39
	LSD_0.05_	173.8	136.8	104.2	
Combined over 2011 and 2012	qGY_2.1_	1171.3	968.9	753	20.8
	LSD_0.05_	149.0	122.0	115.4	
	qGY_8.1_	1294.6	1022.1	753	26.6
	LSD_0.05_	160.7	125.2	115.4	
	qGY_2.1_ and qGY_8.1_	1408.7	1022.2	753	37.8
	LSD_0.05_	184.6	138.9	115.4	
HKR47 × MAS26					
2011	qGY_2.2_	1958	1436	820	36
	LSD_0.05_	215.4	166.8	113.8	
2012	qGY_2.2_	1617	1302	747	24
	LSD_0.05_	183.6	144.8	101.2	
Combined over 2011 and 2012	qGY_2.2_	1788	1369	784	30.6
	LSD_0.05_	197.0	151.8	107.4	

## Discussion

QTL mapping of drought resistance traits in the background of locally adapted *indica*/Basmati rice lines is reported recently from China and India [29]. However, the identification of QTL linked to yield under aerobic conditions in target populations of an environment is critical. Marker-assisted selection using consistent-effect QTL is an efficient approach for developing appropriate aerobic rice varieties. Trait selection is another important concern in molecular breeding for aerobic traits. As in the case of drought, QTL for a number of physiological and morphological traits have been identified but they have limited implications in breeding drought-tolerant rice varieties. Large-effect QTL for grain yield under drought have been recently identified [21,22] and their successful introgression has established a yield advantage under drought [30]. For developing aerobic rice with high yield potential, three QTL with a large effect on grain yield under aerobic conditions have been identified in our study.

The study also identified large-effect QTL for different root traits that may increase plant ability to uptake nutrients under aerobic conditions. Reduced nutrient uptake, especially of nitrogen and phosphorus under aerobic conditions vis-à-vis flooded conditions, has been the most important factor for lower yield in aerobic systems than in flooded systems of rice cultivation. The identification of such root-related traits and QTL associated with these root traits that increase nutrient uptake under aerobic conditions can help develop aerobic rice varieties with high yield potential.

The root system is considered as one important component for solving the problem of water scarcity. The improvement of upland rice through a deeper root system is considered to be a promising way to increase water uptake, reduce lower canopy temperature, increase stomatal conductance, and ultimately increase grain yield under water-stress conditions. A deeper and thickened root system having large xylem vessels has been shown to allow upland rice varieties to extract more water from the soil, resulting in a higher yield potential under water-scarce conditions [31]. Such root traits are highly likely to have a positive effect on increasing the adaptation of rice genotypes to aerobic conditions. Growth of the rice root, in terms of total dry matter, maximum root depth, root length density, root number, and root volume, increases until flowering stage and then decreases sharply to maturity [32]. Kawata and Soejima [33] indicated that roots produced after flowering may play an important role during the grain-filling period that ultimately leads to an increase in yield. Root traits are critical for increasing yield under soil-related stresses.

Correlation analysis was carried out to identify how root morphological characters influence the grain yield and yield morphological traits under aerobic conditions. Our study identified a significant and positive correlation between some of the root traits (root number, root volume, root thickness and dry root weight) and yield under aerobic conditions, indicating the role of root traits for improving yield under aerobic situations possibly through improved water and nutrient uptake. This shows that a well developed root system will help the plant in maintaining high plant water status which ultimately leads to increase in yield potential under water deficit conditions.

**Figure 5:**
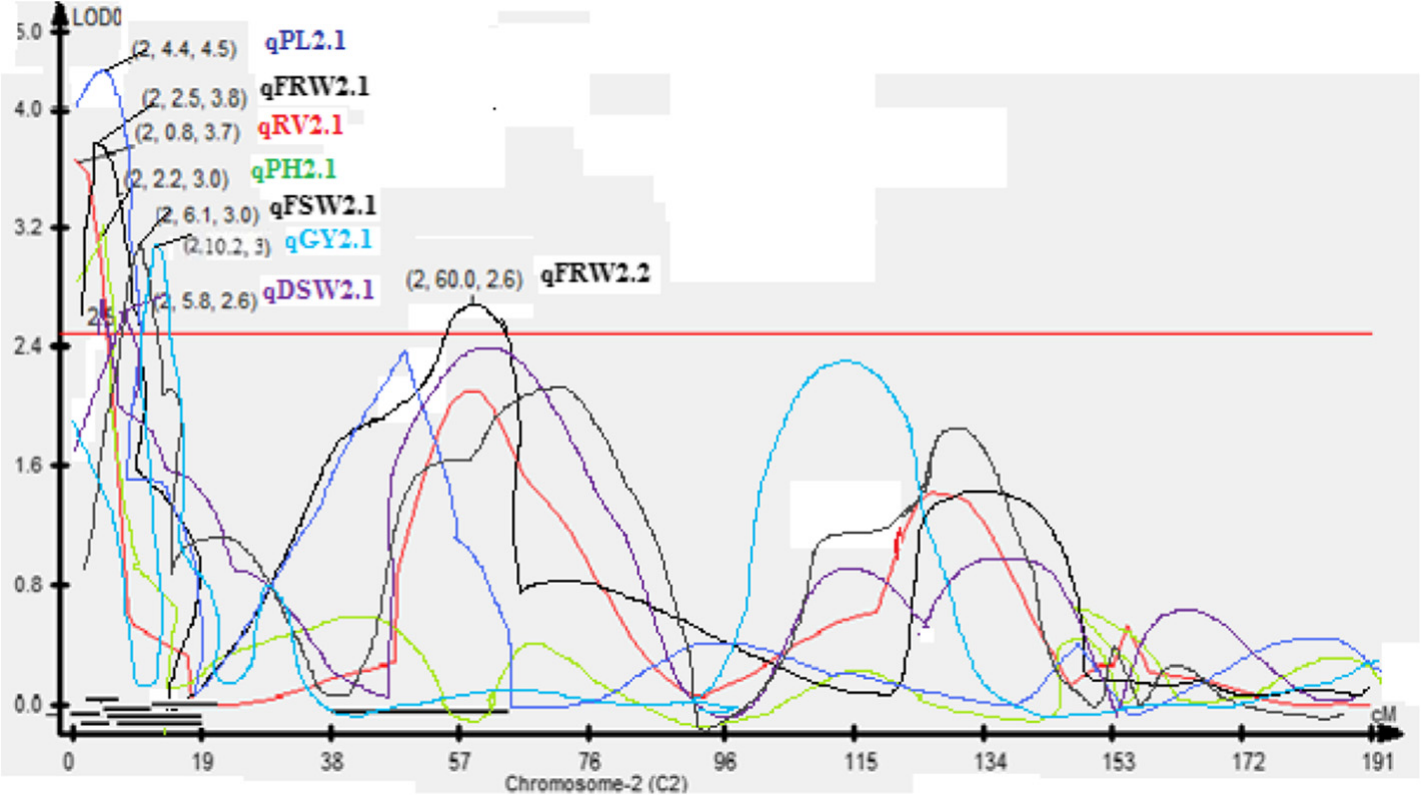
QTL likelihood curves of LOD score of a segment of 10.2 cM on chromosome 2 in MASARB25 × Pusa Basmati 1460 population.

Correlated characters are of interest for three main reasons: in connection with the genetic causes of correlation through the pleiotropic action of genes, to know how selection for one character will cause a simultaneous change in other characters, and to determine the relationship between character and fitness. In classical quantitative genetics, trait correlations are attributed to the effect of pleiotropy or very close linkage of genes [34] but unwanted traits might also be selected during MAS because of the co-localization of QTL. The magnitude and direction of influence of these loci on the different phenotypes will markedly affect the utility of such loci in selection for simultaneous improvement of these traits [35]. In our study, on chromosome 2 in a region of 0-10.2 cM, seven QTL were reported to be significantly associated with seven different root traits and agronomically important traits (RV, FRW, FSW, DSW, PH, PL, and GY) in the case of MASARB25 × Pusa Basmati 1460 population (Figure 5). Two QTL, qGY_2.1_ in the 10-cM region between RM7562 and RM279 on chromosome 2 in MASARB25 × Pusa Basmati 1460 population and qGY_2.2_ in the 118.5-cM region between RM475 and RM526 on chromosome 2 in HKR47 × MAS26, for grain yield under aerobic conditions were identified. qGY_2.1_ and qGY_8.1_ in MASARB25 × Pusa Basmati 1460 showed a stable high over two different years and combined over two years individually, and an increased stable effect over two different years and combined over two years (Table 6). Similarly, in HKR47 × MAS26, qGY_2.2_ showed a stable high effect over individual years and combined over two years. These QTL were found to be adjacent to the earlier reported QTL qDTY_2.2_ and qDTY_2.3_[21,36] for grain yield under drought stress. Although the effect of the two identified QTL needs to be validated in different genetic backgrounds, under the present scenario, successful introgression of the identified QTL following marker-assisted backcrossing can be used to improve the popular varieties Pusa Basmati 1460 and HKR47.

Similarly, QTL for root length within the qGY_2.1_ region [37] and for root thickness adjacent to qGY_2.2_[38] have been reported. Coexisting chromosomal regions/loci governing different traits provide a unique opportunity for breeders to introgress such regions together as a unit into high-yielding lowland varieties through MAS/MAB and to develop cultivars possessing increased adaptation to aerobic conditions.

Two QTL, qRL_8.2_ in both mapping populations and qRL_8.1_ in MASARB25 × Pusa Basmati 1460, for root length have been reported in our investigation. Qu et al. [39] reported QTL for fresh root weight and root number coinciding with qRL_8.2_ linked to marker RM331. Studies also compiled numerous earlier reports of QTL for root length [40], root thickness, and root number [41] adjacent to or coinciding with qRL_9.1_. In HKR47 × MAS26 mapping population, qRT_1.1_ was reported for root thickness with an LOD of 4.4 and R^2^ value of 38.4%. These QTL may confer a grain yield advantage under direct-seeded conditions and this is supported by the earlier reported large-effect consistent QTL (qDTY_1.1_) for grain yield under drought [42] in the region of qRT_1.1_.

**Figure 6:**
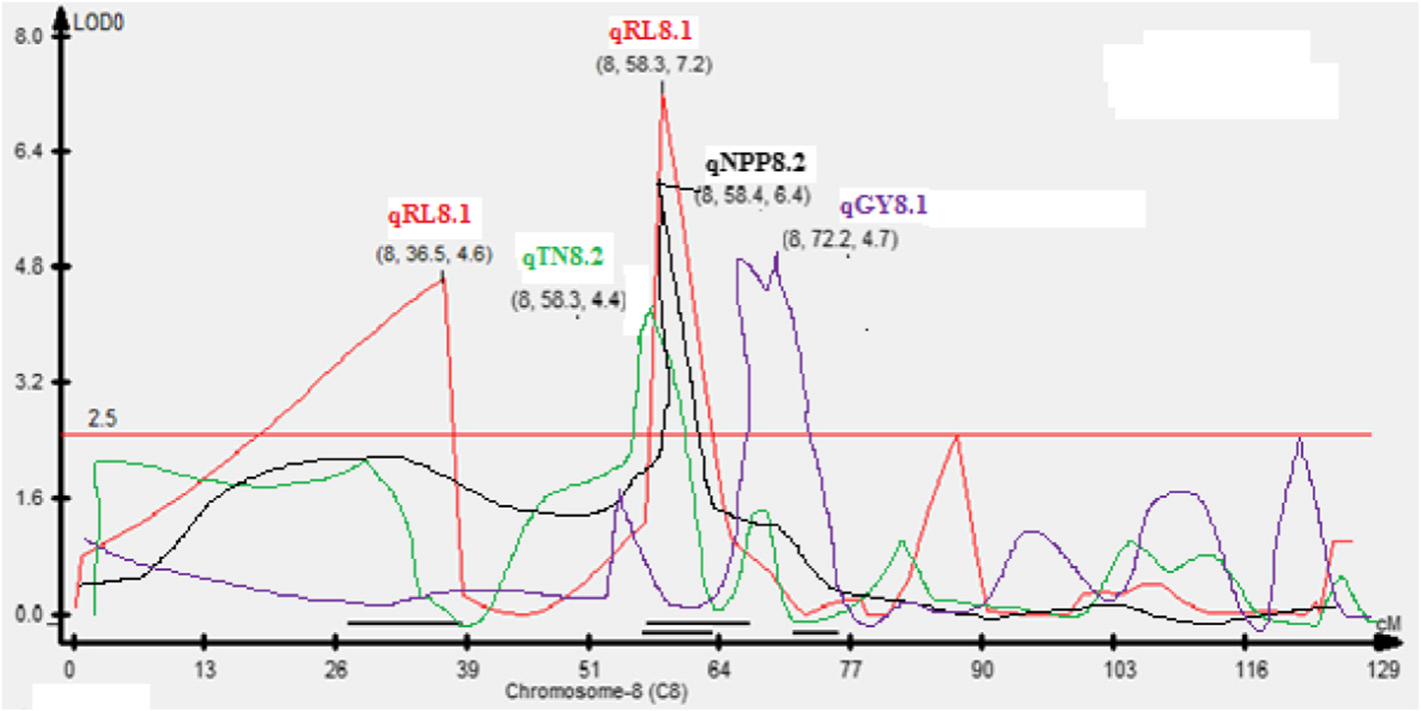
QTL likelihood curves of LOD score of coexisting qtl on chromosome 8 in MASARB25 × Pusa Basmati 1460 population.

Under dry direct-seeded conditions, the QTL peak (qGY_8.1_) for grain yield was seen in MASARB25 × Pusa Basmati 1460 at RM339 with an R^2^ value of 34%. At the same position and linked to the same marker (RM339), qDTY_8.1_ in Basmati334/Swarna was reported by Vikram et al. [43] for grain yield under drought. Hanamaratti, [44] reported RM339 on chromosome 8 associated with relative yield and drought susceptibility index in IR64 × Binam-derived NILs under drought stress. Adjacent to the QTL qGY_8.1_ within a region of 14.4 cM, other QTL (qTN_8.2_, qNPP_8.2_, and qRL_8.2_) have been reported (Figure 6). In a segment of 21.7 cM on chromosome 8 (101.5-123.2 cM), QTL for DRW, TN, NPP, PL, NSP, PH, and GY were also reported in our study. The effect of these regions on a number of traits that is likely to impart improved aerobic adaptation. GY strongly suggests the presence of more than one gene within these QTL affecting a wide range of traits. These genes conferring a GY advantage under aerobic adaptation may have undergone strong natural selection to stay together in the course of evolution. These sub-QTL with a discernible phenotypic effect on GY may affect the same/different physiological traits in response to different severities of stress, leading to a GY advantage.

QTL qPL_12.1_ (RM28048) identified in our study in HKR47 × MAS26 mapping population showed an effect on a yield-attributing trait (i.e., panicle length) under dry direct-seeded conditions. Bernier et al. [21,45] reported QTL qtl_12.1_ linked to the same marker. Bernier et al. [45] and Dixit et al. [36] showed a consistent large effect on grain yield under drought over a wide range of environments in the Philippines and eastern India. This confirms that this QTL is effective not only at IRRI, where it was initially detected, but also in at least a part of the target environment of eastern and northern India, where improved drought resistance in upland rice cultivars is an important breeding objective. This is also supported by the study of Bernier et al. [21] that mentioned that this locus is associated with a low QTL × environment interaction under severely stressed conditions, which is one of the two major requirements for the use of a QTL in MAS. The QTL for biomass, harvest index, days to flower, plant height, flowering delay, drought response index, and panicle number under stress were mapped in the same region. qtl_12.1_ also influences water uptake under upland stress and increases harvest index [21].

**Figure 7:**
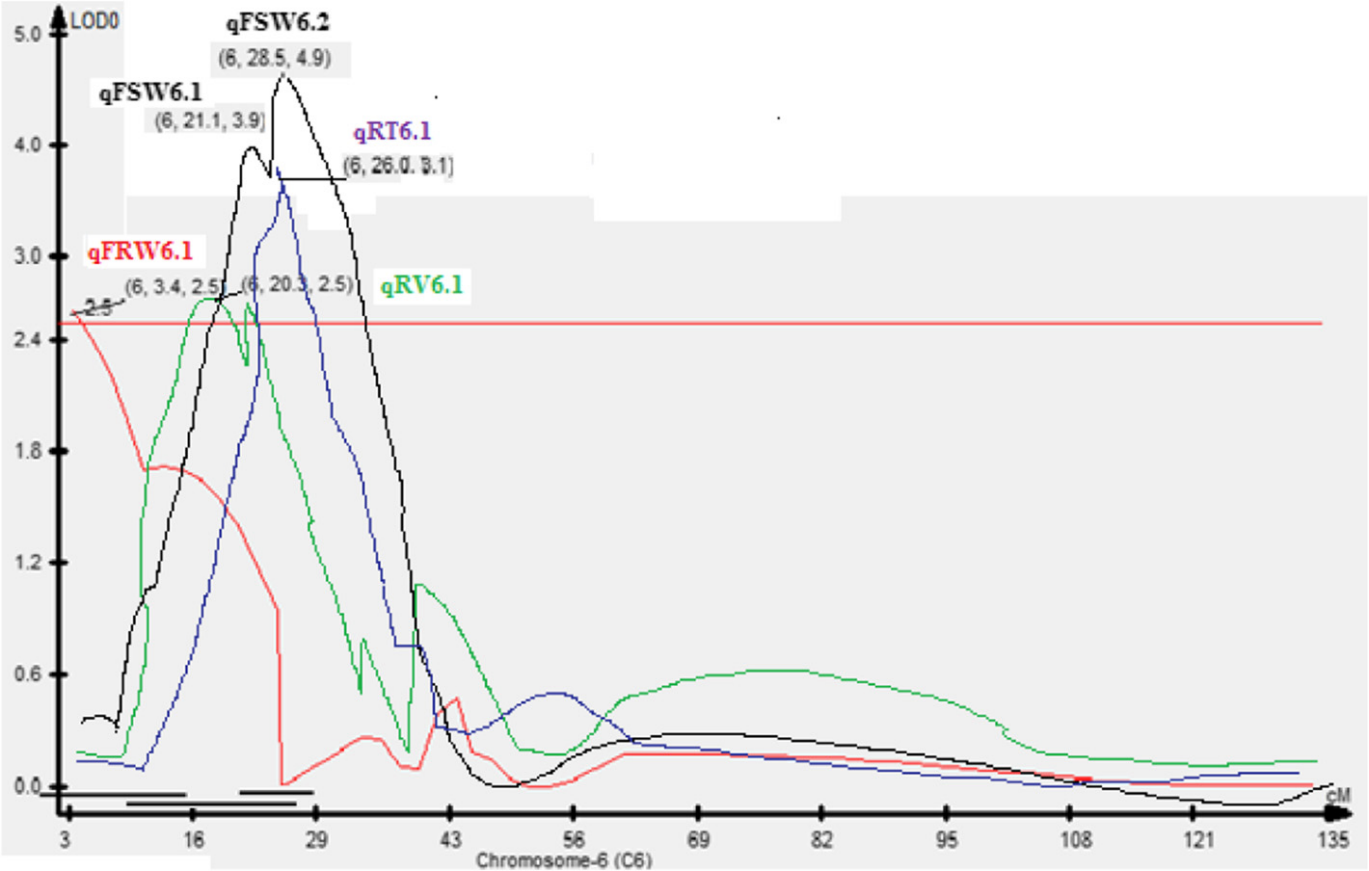
QTL likelihood curves of LOD score of coexisting qtl on chromosome 6 in MASARB25 × Pusa Basmati 1460 population.

In our study, we reported a 25.1-cM segment between RM589 and RM314 on chromosome 6 affecting different root (RV, RT, and FRW) and shoot (FSW and DSW) traits under aerobic conditions in the case of MASARB25 × Pusa Basmati 1460 mapping population (Figure 7). In this region, a large-effect QTL (qDTY_6.1_) associated with grain yield in aerobic environments was identified in a total of 20 hydrological environments over a period of five seasons and in five populations in three genetic backgrounds using bulk-segregant analysis [22]. Co-localization of qDTY_6.1_ with a region identified to govern several root and shoot traits is an implication that this is an important region for improving yield under aerobic conditions.

Our study identified a number of lines with higher yield under aerobic conditions, higher root length and dry biomass, GY, and length/breadth ratio, and with Pusa Basmati 1460-specific alleles in homozygous or heterozygous condition at the *BAD2* locus (Table 7). These lines will serve as novel materials for the selection of stable aerobic Basmati rice varieties.

**Table 7:** Values of mean yield and mean root traits of selected promising lines under aerobic conditions

**Population**	**Pusa Basmati 1460**	**MASARB25**	**MASARB25 × Pusa Basmati 1460**	**HKR47**	**MAS26**	**HKR47 × MAS26**
No. of promising lines selected			42			52
GY (kg ha^-1^)	717	852	1889.3	747	932	2346.2
LSD_0.05_	104.2	133.3	234.1	101.2	156.7	198.2
RL	20.22	27.22	29.93	20.00	29.45	33.42
LSD_0.05_	0.996	1.175	1.112	1.332	1.545	1.443
RV	13.0	16.0	18.8	7.1	8.2	8.8
LSD_0.05_	0.621	0.545	0.775	0.399	0.452	0.576
RN	88	102	109	85	92	97
LSD_0.05_	1.994	2.223	2.899	1.132	1.459	1.886
RT	2.5	3.2	3.6	2.0	2.6	3.1
LSD_0.05_	0.445	0.776	0.356	0.778	0.597	0.662
FRW	3.45	4.67	5.02	2. 42	3.12	4.01
LSD_0.05_	0.532	0.445	0.668	0.448	0.439	0.665
DRW	1.02	1.34	1.57	1.12	0.77	1.34
LSD_0.05_	0.554	0.454	0.379	0.229	0.236	0.332
Aroma	+ +	- -	+ +, + -	- -	- -	- -

*GY*: Grain yield, *RL*: Root length, *RN*: Root number, *RV*: Root volume, *RT*: Root thickness, *FRW*: Fresh root weight, *DRW*: Dry root weight, *++* Aromatic (homozygous), *+ -* : Aromatic (heterozygous), *- -*: Non-aromatic (homozygous).

## Conclusions

Our study identified a total of 35 QTL associated with 14 traits on chromosomes 1, 2, 5, 6, 8, 9, and 11 in MASARB25 × Pusa Basmati 1460 population and 14 QTL associated with nine traits on chromosomes 1, 2, 8, 9, 10, 11, and 12 in HKR47 × MAS26-derived population. These identified QTL included three large-effect stable QTL for increased yield under aerobic conditions and QTL for several root-related traits likely to increase water and nutrient uptake under aerobic conditions. A mechanism associated with higher yield of promising lines under dry direct-seeded conditions, indicating relocation of resources during grain filling, is suggested by coexisting QTL for root and yield-attributing traits. Results from our study suggest that reported QTL are complex loci where multiple genes may be working independently or in coordination with each other, leading to an increase in GY under drought. Coexisting chromosomal regions/loci governing different traits for aerobic adaptation provide a unique opportunity for breeders to introgress such regions together as a unit into high-yielding drought-susceptible varieties through MAS/MAB and to develop cultivars possessing increased tolerance of varying stress severities.

## Abbreviations

cm: Centimeter; CTAB: Cetyltrimethyl ammonium bromide; DNA: Deoxyribonucleic acid; DPE: Direction of phenotypic effect; DRW: Dry root weight; DSW: Dry shoot weight; FRW: Fresh root weight; FSW: Fresh shoot weight; g: Gram; 1,000 GW: 1,000-grain weight; GY: Grain yield; IRRI: International Rice Research Institute; Kg ha-1: Kilogram per hectare; L/B: Length/breadth ratio; LOD: Logarithm of odds; LR: Likelihood ratio; MAB: Marker-assisted breeding; MAS: Marker-assisted selection; ml: Milliliter; mm: Millimeter; NPK: Nitrogen, phosphorus, and potassium; P/P: Number of panicles plant^-1^; PL: Panicle length; PH: Plant height; PAGE: Polyacrylamide gel electrophorosis; PCR: Polymerase chain reaction; RIL: Recombinant inbred line; R2: Percent phenotypic variance; RL: Root length; RN: Root number, RV, Root volume; RT: Root thickness; S/P: Seeds panicle^-1^; TN: Effective number of tillers plant^-1^.

## Competing interests

In the past five years have you received reimbursements, fees, funding, or salary from an organization that may in any way gain or lose financially from the publication of this manuscript, either now or in the future? Is such an organization financing this manuscript (including the article-processing charge)? If so, please specify.–No

Do you hold any stocks or shares in an organization that may in any way gain or lose financially from the publication of this manuscript, either now or in the future? If so, please specify.–No

Do you hold or are you currently applying for any patents relating to the content of the manuscript? Have you received reimbursements, fees, funding, or salary from an organization that holds or has applied for patents relating to the content of the manuscript? If so, please specify.–No

Do you have any other financial competing interests? If so, please specify.–No

Non-financial competing interests

Are there any non-financial competing interests (political, personal, religious, ideological, academic, intellectual, commercial or any other) to declare in relation to this manuscript? If so, please specify.–No

## Authors’ contributions

NS was involved in the conception of the experiment, analysis, interpretation of the data, and drafting the article and final approval of the version to be published; SJ was involved with conception and design of the experiment, revising manuscript content critically and final approval of the version to be published; AK was involved with the analysis, interpretation of the data, in the critical revision of the manuscript and final approval of the version to be published; BSM was involved in the conception of the experiment, in the critical revision of the manuscript and final approval of the version to be published; RKJ was involved in the design of the experiment, the critical revision of the manuscript and final approval of the version to be published.

## Supplementary Material

Additional file 1: Figure S1 QTL associated with agronomic and aerobic root traits of MASARB25 × Pusa Basmati 1460 population. **Figure S2.** QTL associated with agronomic and aerobic root traits of HKR47 × MAS26 population. Click here for file
