# Supplementary figures and images for: Genetic variation, linkage mapping of QTL and correlation studies for yield, root, and agronomic traits for aerobic adaptation
Source: BMC Genet. 2013 Oct 29;14:104. doi: 10.1186/1471-2156-14-104 (PMC4231467; doi:10.1186/1471-2156-14-104)

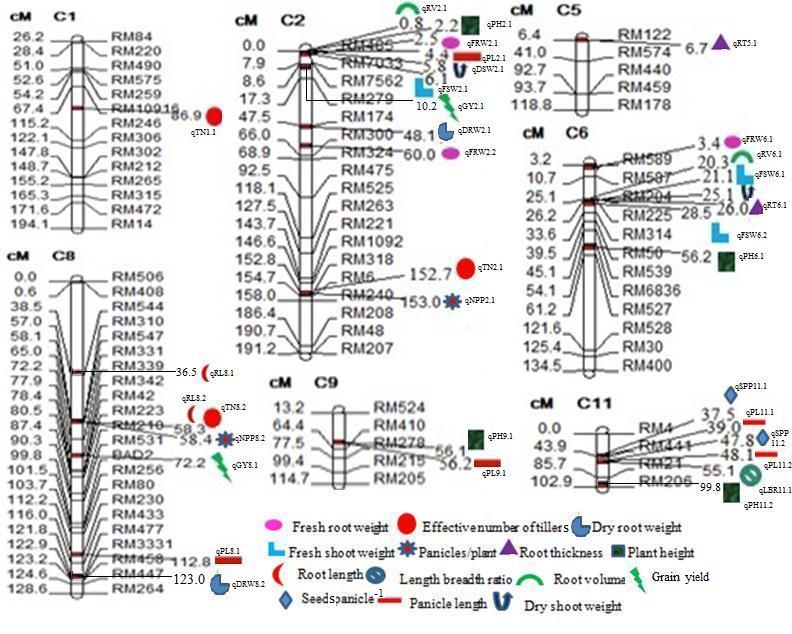


Supplementary Figure 1


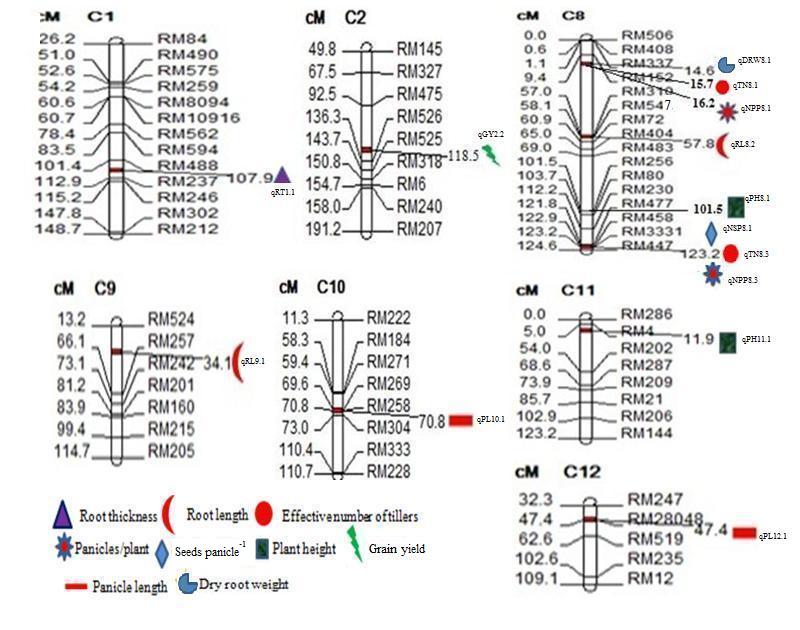


Supplementary Figure 2

Supplement: Additional file 1: Figure S1 — QTL associated with agronomic and aerobic root traits of MASARB25 × Pusa Basmati 1460 population. Figure S2. QTL associated with agronomic and aerobic root traits of HKR47 × MAS26 population. [file 1471-2156-14-104-S1.doc]
